# Air exchange rates and advection–diffusion of CO_2_ and aerosols in a route bus for evaluation of infection risk

**DOI:** 10.1111/ina.13019

**Published:** 2022-03-20

**Authors:** Naohide Shinohara, Koichi Tatsu, Naoki Kagi, Hoon Kim, Jun Sakaguchi, Isamu Ogura, Yoshiko Murashima, Hiromu Sakurai, Wataru Naito

**Affiliations:** ^1^ National Institute of Advanced Industrial Science and Technology (AIST) Tsukuba Ibaraki Japan; ^2^ Isuzu Motors Ltd Fujisawa Kanagawa Japan; ^3^ Tokyo Institute of Technology Meguro‐ku, Tokyo Japan; ^4^ National Institute of Public Health Wako Saitama Japan; ^5^ University of Niigata Prefecture Niigata‐City, Niigarta Japan

**Keywords:** airborne transmission, commuter, SARS‐CoV‐2, ventilation, window‐opening

## Abstract

As COVID‐19 continues to spread, infection risk on public transport is concerning. Air exchange rates (ACH) and advection–diffusion of CO_2_ and particles were determined in a route bus to evaluate the infection risk. ACH increased with bus speed whether windows were open or closed, and ACH were greater when more windows were open. With two open windows, ACH was greater when a front and rear window were open than when two rear windows were open. With both front and rear ventilation fans set to exhaust, ACH was more than double that when both were set to supply. With air conditioning (AC) off, CO_2_ and particles spread proportionally at the same rate from a source, whereas with the AC on, the spread rate of particles was about half that of CO_2_, because particles might be trapped by a prefilter on the AC unit. Infection risk can be reduced by equipping AC unit with an appropriate filter. Calculations with a modified Wells–Riley equation showed that average infection risk was reduced by 92% in the moving bus with windows open comparing to with windows closed. When the bus was moving with windows closed, exhaust fan operation reduced the average risk by 35%.

**Practical Implications**

- Air exchange rates increased with vehicle speed and the number of open windows.
- In the moving bus with two windows open, ACH were greater when front and rear windows were open.
- The spread of particles was about the same as that of CO_2_ when the AC was off, and but half that of CO_2_ when the AC on.
- In the moving bus, open windows and exhaust fan operation reduced the average infection risk by 92% and 35%, respectively.

## 1 INTRODUCTION

Since the end of 2019, the infection numbers of the novel coronavirus disease COVID‐19 have been expanding around the world.^1^, ^2^ The COVID‐19 infection risk on public transport such as trains and buses, which are used by an unspecified number of people, is a matter of great public concern. In Japan in 2010, 15 million people per day commuted to and from work or school using trains, and 4.2 million commuted daily on buses.^3^ Reducing the risk of infection on public transport is a socially important issue, and the development of effective and specific measures to reduce the infection risk is important to government, transport operators, and commuters.^4^

Generally, three modes were responsible for the infection of virus, including droplet transmission, airborne transmission, and contact transmission. Regarding infection by SARS‐CoV‐2, the virus responsible for COVID‐19 and the relative contributions of transmission routes remain uncertain and it depends on the situation, although the possibility of contact transmission is not high.^5^ However, the importance of airborne transmission has recently been widely suggested.^6^, ^7^, ^8^, ^9^, ^10^ To properly assess the risk of airborne transmission and to determine countermeasures that should be taken, it is necessary to understand ventilation conditions and airflow and particle behaviors in the space of interest.

Many studies have investigated airflow and particle behaviors in mobile vehicles transporting passengers, but the majority of these have been numerical simulations.^11^, ^12^, ^13^ For example, although an aircraft cabin is an enclosed space, approximately 50% of the cabin air is derived from outdoor air; further, all the cabin air is exchanged with outdoor air every 3–4 min, and the recirculated air is cleaned by passing through a HEPA filter, which can remove 99.97% of 0.1–0.3 μm particles.^14^, ^15^ Compared to an aircraft cabin, in buses the performance of ventilation fans and air conditioning filters has not been well investigated. Although computational fluid dynamics (CFD) analyses^16^, ^17^ and particle measurements^17^ have been conducted to investigate the diffusion of droplets in a stationary bus, no studies have obtained empirical evidence to evaluate airflow in actual moving buses.

The SARS‐CoV‐2 outbreaks in tourist buses have been reported,^18^ but no such outbreaks have been reported for route buses. The arrangement of the doors and windows and vehicle operation of tourist buses are quite different from those of route buses.

In a case–control study conducted during influenza season, Troko et al^19^ found a statistically significant association between acute respiratory infection and bus use. Yang et al^20^ conducted a CFD analysis to evaluate the behavior of large droplets (10 and 50 μm) in a bus; they found that a backward in‐cabin airflow and passenger sitting in nonadjacent seats reduced infection risk. Zhu et al,^21^ who conducted a CFD analysis of airflow, showed that the risk of influenza infection in buses is higher for passengers sitting between the infected person and the exhaust vent than for passengers standing in the front cabin section, but it can be reduced by the use of displacement ventilation.

Dai and Zhao^22^ used the Wells–Riley equation to calculate the risk of COVID‐19 transmission in various types of locations; they estimated the quanta emission rate, *q*, of COVID‐19 to range from 14 to 48/h by using the basic reproductive number (*R*_0_) for COVID‐19 (2.0–2.5) and the fitted curve between known *q* values and *R*_0_ values for other airborne transmitted infectious diseases reported by previous studies. For *q* of 14–48/h, they reported that to keep the infection risk at less than 0.1% in a bus with one infected person, the air exchange rate (ACH: air changes per hour) must be at least 7.0–24/h if passengers wear masks, and 27–93/h if they do not. Rim et al^23^ reported that the ACH in a bus, based on the exponential decay of the SF_6_ concentration, were determined to be 2.60–4.55/h during typical operation. In Japan, opening the windows to improve ventilation is recommended in buses,^24^, ^25^ but measurements of ACH inside a bus when the windows are open have not been reported.

Shinohara et al.^26^ estimated the risk of airborne COVID‐19 transmission based on measured ACH in subway trains, but they did not measure the spread of aerosol particles such as smaller droplets and droplet nuclei. Moreover, unlike subway trains, buses are equipped with ventilation fans, which may lead to different ventilation behavior.

In this study, to obtain empirical data on the effectiveness of countermeasures that would contribute to infection control in route buses, ACH in a bus were determined under various conditions by using the CO_2_ decay method. In addition, to simulate the advection–diffusion of droplet nuclei from an infected individual, CO_2_ and particles were emitted at one point in the bus, and the spread of gaseous CO_2_ and particles in the bus from that point was measured and evaluated. Then, the infection risk in the bus was evaluated and discussed.

## 2 MATERIALS AND METHODS

### 2.1 Survey period and bus characteristics

During 1–5 and 28–30 August 2020, ACH were determined in a large route bus (Isuzu LV290Q1; Figure S1). Stationary tests were conducted in a car barn (12 m width, 22 m length, 6.0 m height) at the Fujisawa Plant of Isuzu Motors Ltd. (Fujisawa, Kanagawa, Japan) in which the effect of wind outside the bus was negligible and in an outdoor open space at AIST Tsukuba West (Tsukuba, Ibaraki, Japan). Moving tests were conducted on a peripheral road at AIST Tsukuba Central (approximately 3 km around, Tsukuba, Ibaraki, Japan).

**FIGURE 1:**
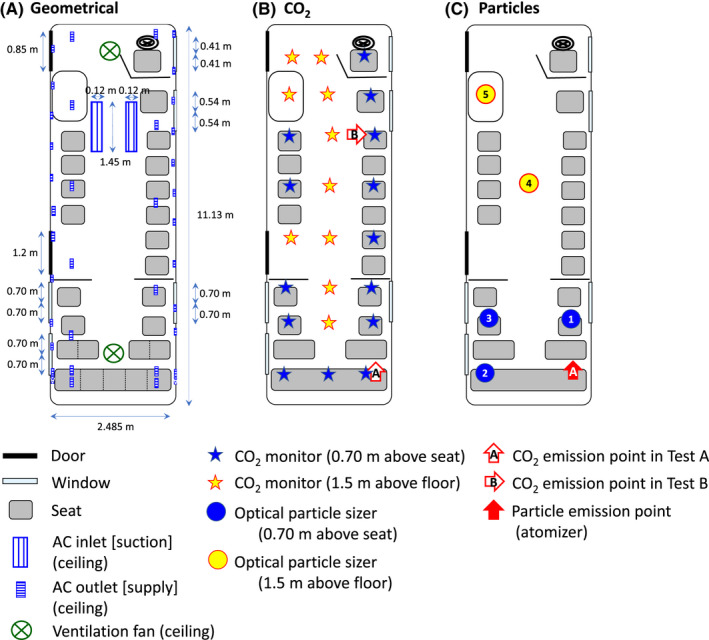
(A) Interior configuration and dimensions of the commuter bus; (B) CO_2_ and (C) particle emission and monitoring points in the bus

Figure 1A shows the interior configuration and dimensions of the bus. The inside of the bus was 11 m long and 2.5 m wide (total interior volume: 50 m^3^). It contained 25 seats (including the driver's seat) and a total of 5 openable windows (3 windows, 0.70 m × 0.35 m × 2 panes; 1 window, 0.54 m × 0.35 m × 2; 1 window, 0.41 m × 0.77 m × 2), 3 on the right side and 2 on the left side of the bus (Figure S2). Two doors were located on the left side of the bus. Two ventilation fans, both of which could be set to either supply or exhaust air, were located in the center of the ceiling near the front and toward the back of the bus (Figure S2). Two air conditioner (AC) suction inlets (return vents) were located in the ceiling at the center front of the bus, and 38 AC outlets (supply vents) were located in the ceiling above passenger seats (Figure S2). The total flow rate of the AC in the bus was 3850 m^3^/h, which means that air equivalent to the room volume can be circulated 77 times in an hour. A coarse dust filter was set in the AC unit.

### 2.2 Determination of air exchange rates

ACH were determined by the CO_2_ decay method,^27^, ^28^ which is appropriate for obtaining short‐term ACH in an unoccupied space. After CO_2_ emission from gas cylinders in the bus, in‐bus air was agitated by four circulators (EAC‐23‐W, Iris Ohyama Inc., Sendai, Japan) and two blowers (Earthman BW‐144LiAX, Takagi Co., Ltd., Kitakyushu, Japan) until the CO_2_ concentration reached equilibrium across all sampling points. The in‐bus CO_2_ concentration was monitored with CO_2_/humidity/temperature data recorders (MCH‐383SD, Lutron Electronic Enterprise Co., Taipei, Taiwan) every 10 s for 15 min to 1 h at 24 points in the bus (Figure 1B); 10 monitoring points were at a height of 1.5 m (to estimate the exposure of standing passengers) and 14 were at a height of 0.7 m above the seat level (sitting passengers) to match the height of the passenger's mouth. The outdoor CO_2_ concentration was measured with the same CO_2_ instruments at the same time at a height of 1.5 m above the ground within 100 m of the bus and out of direct sunlight. In‐bus and outdoor temperatures were also measured with the same instruments.

ACH were calculated by fitting an exponential function to differences in the monitored CO_2_ concentrations between indoor and outdoor environments. Before the survey, all of the CO_2_ recorders were calibrated to several different concentrations of CO_2_ gas, including atmospheric level, measured by a previously calibrated CO_2_ monitor (Model 2211, Kanomax Japan, Inc., Japan).

To evaluate differences in the ACH due to different vehicle speeds, AC operation, the presence or absence of passengers, operation of ventilation fans, and open or closed windows, the CO_2_ concentration decay was determined both while the bus was stationary (0 km/h) and while it was moving at 10, 20, or 30 km/h, with the AC off or on, with or without passengers (mannequins), with the ventilation fans off or on, and with the windows closed or open (Table S1). All test conditions were tested once. The upper limit of vehicle speed was set at 30 km/h to simulate rush hour traffic, and speeds of 10 and 20 km/h were considered to simulate the slower vehicle speeds seen in large cities since the average moving speed of vehicles during rush hour was 14.3–15.4 km/h in Tokyo and 16.9–35.6 km/h in whole Japan 2015.^29^ To represent passengers, 75 mannequins (25 sitting mannequins and 50 standing mannequins) made of resin without heat were placed in the bus to simulate a full cabin. While the AC was on, the temperature was set to 26°C. In the open windows test, both sides of each of the five windows were opened to the maximum amount (Figure S3). In addition, we conducted a test without mannequins to simulate typical operation of a route bus; in this test, the bus repeatedly moved 500 m at 30 km/h and then stopped and opened the door for 20 s (hereafter, the stop & go test).

To evaluate ACH when only some windows were open, the CO_2_ concentration decay was measured with all five windows open (either fully or half open), with two windows open (front right and rear left), with two windows open (front right and rear right), and with two windows open (rear right and rear left).

To evaluate the effect of ventilation fan operation, the CO_2_ concentration decay was measured under four different conditions by separately setting the front and rear ventilation fans to either exhaust or supply mode.

Moving tests were conducted at constant speeds of 10, 20, and 30 km/h and were repeated for two or three laps around the 3 km of peripheral road. Other conditions of the moving tests were basically the same as those of the stationary tests.

In Tsukuba, temperatures (average ± SD) on 1–5 August 2020 during the stationary and moving tests with mannequins were 27 ± 1.8°C inside the bus with the AC on, 31 ± 2.4°C inside the bus with the AC off, and 36 ± 3.5°C outside the bus. Temperatures on 28–30 August 2020 during the stationary and moving tests without mannequins were 26 ± 2.3°C inside the bus with the AC on, 34 ± 3.7°C inside the bus with the AC off, and 34 ± 1.5°C outside the bus. The temperature inside and outside the vehicle in Fujisawa, where stationary tests were conducted in a car depot, was 25 ± 1.5°C inside the vehicle and 23 ± 0.40°C outside the vehicle.

### 2.3 Advection–diffusion of artificial droplet nuclei

To evaluate the advection–diffusion of droplet nuclei from an infected individual in the bus, CO_2_ or particles were emitted at a constant volume flow rate from a single location and measured at 24 (CO_2_) and 5 points (particles). For CO_2_ (Figure 1B), the emission point was either at location A at the rearmost right‐side seat, simulating a sitting passenger facing the front, or at location B in the middle near the second frontmost right‐side seat, simulating a standing passenger facing the window. For particles, the emission point was only at location A in Figure 1C. Every test condition is listed in (Table S2).

**FIGURE 2:**
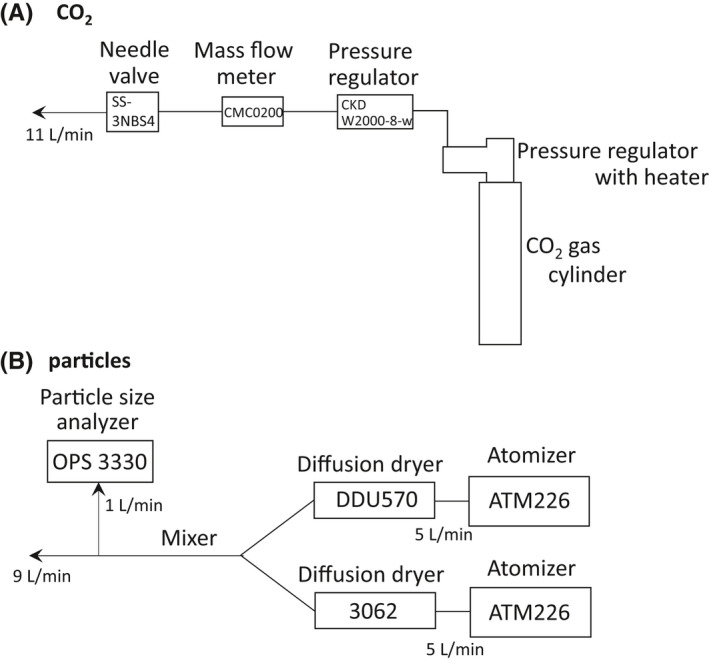
(A) CO_2_ and (B) particle emission systems

CO_2_ was emitted at a flow rate of approximately 11 L/min (linear airflow rate, approximately 3.0 m/s; diameter of outlet port, 9 mm) from gas cylinders equipped with a primary pressure regulator with heater (YR‐510F‐2, Yamatosangyo Co. Ltd., Osaka, Japan), a secondary pressure regulator (CKD W2000‐8‐w, CKD Co. Ltd., Aichi, Japan), and a bonnet needle valve (SS‐3NBS4, Swagelok, Ohio, USA; Figure 2A). This CO_2_ emission rate corresponds to the inhalation rate (~10 L/min) of a sitting person (AIST, 2007). The emission rate was monitored by a mass flow meter (CMC0200, Azbil Co. Ltd., Tokyo, Japan) equipped with a data logger. CO_2_ data recorders (MCH‐383SD) were used to continuously measure CO_2_ (every 10 s) and the CO_2_ decay measurement (Figure 1B).

Polystyrene latex particles with a diameter of 1.3 μm (Research Particle 5130A, Thermo Fisher Scientific; Massachusetts, USA) were emitted by two atomizers (ATM 226, Topas GmbH, Dresden, Germany) with diffusion dryers (DDU 570/H, Topas GmbH; 3062, TSI Incorporated, Minnesota, USA; Figure 2B). This particle diameter corresponds to the diameter of aerosol particles such as droplet nuclei.^30^ The flow rate of each atomizer was set to approximately 5.0 L/min. Particle‐containing air from the two atomizers was combined by using conductive tubing and a Y‐shaped connecting tube and then mixed with a static mixer (T8‐15R, Noritake Co., Limited, Aichi, Japan). The total flow rate, which was measured with a mass flow meter (4143, TSI Incorporated, Minnesota, USA) at the start and end of emission, was approximately 9.0 L/min (linear flow rate 3.1 m/s; diameter of outlet port, 7.9 mm), similar to that of CO_2_. Optical particle sizers (OPSs; OPS3330, TSI Incorporated, Minnesota, USA) were used to continuously measure particles (measurement interval, 10 s; particle size range, 0.9–2.2 µm) at the source (after particles from the two atomizers were mixed) and at five other locations in the bus (1.5 m from the floor in the standing area or 0.70 m from the floor in the seating area; Figure 1C).

In the laboratory before the survey, the line from an atomizer was divided six ways and simultaneously connected to the six OPSs, and the emitted particles were measured. The relative values were used to correct for differences among the OPSs.

In the first test, CO_2_ and particles were emitted at location A (Figure 1B and C) for 15 min, and in the second test, CO_2_ was emitted at location B (Figure 1B) for 15 min. Both tests were conducted while the bus was stationary. Other tests were conducted while the bus was moving. At each measurement point, the amount of CO_2_ or particles inhaled relative to the amount of CO_2_ or particles emitted, *P*_CO2_ and *P*_particles_ [%], respectively, was determined based on the assumption that a seated or standing passenger remained at the measurement point for the duration of the test.

(1) $$P_{{\mathrm{CO}}_{2}}=\frac{I\int_{0}^{t}C_{{\mathrm{CO}}_{2}}\left(t\right)dt/1000000}{\int_{0}^{t}E_{{\mathrm{CO}}_{2}}\left(t\right)dt}\times 100$$

(2) $$P_{\mathrm{particles}}=\frac{I\int_{0}^{t}C_{\mathrm{particles}}\left(t\right)dt}{\int_{0}^{t}E_{\mathrm{particles}}\left(t\right)dt}\times 100$$

where *I* [m^3^/h] is the inhalation rate of each passenger, *C*_CO2_(*t*) [ppm] and *C*_particles_(*t*) [particles/m^3^] are the differences in the concentration of CO_2_ and particles, respectively, between in‐bus and background (outdoor) measurements at elapsed time *t* [h], and *E*_CO2_ (*t*) [m^3^/h] and *E*_particles_(*t*) [particles/h] are the CO_2_ and particle emission rates at elapsed time *t*, respectively. The inhalation rate, *I*, was set at 0.60 m^3^/h, assuming a sitting passenger.^31^

## 3 RESULTS

### 3.1 Air exchange rates

**FIGURE 3:**
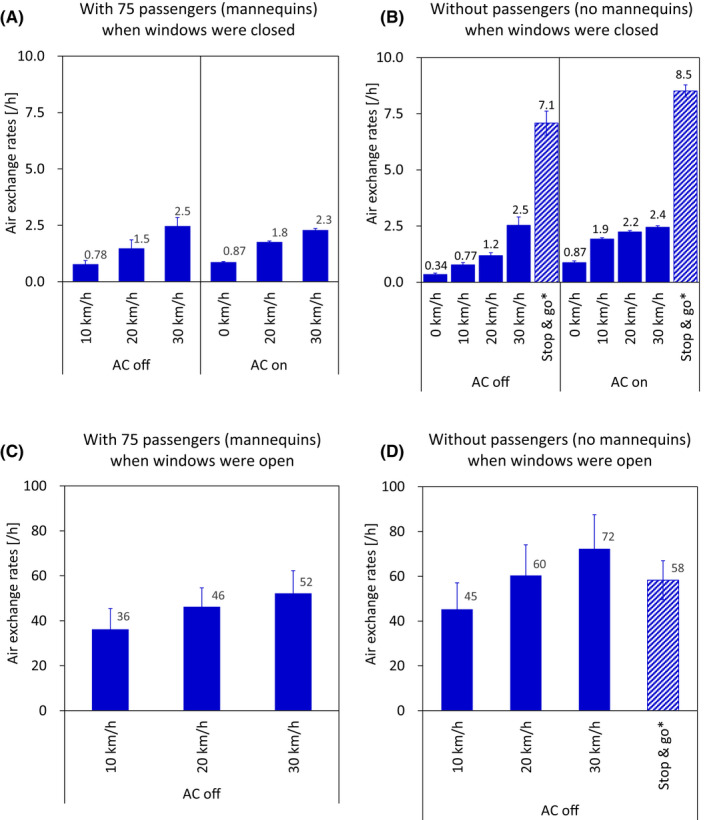
ACH when the bus was moving at different speeds, determined at Tsukuba: (A) 75 passengers (mannequins) and windows closed, (B) no passengers (mannequins) and windows closed, (C) 75 passengers (mannequins) and windows open (5 windows fully opened), (D) no passengers (mannequins) and windows open (5 windows fully opened). * During the stop & go test, the bus repeatedly moved 500 m at 30 km/h and then stopped and opened the door for 20 s

The ACH increased as the speed of the bus increased, regardless of AC operation and whether windows were open or closed (Figure 3A–D). For example, with mannequins, and all windows closed (Figure 3A), the ACH increased from 0.78/h at 10 km/h to 2.5/h at 30 km/h with the AC off and from 0.87/h at 10 km/h to 2.3/h at 30 km/h with the AC on, whereas with mannequins and all windows open (Figure 3C), it increased from 36/h at 10 km/h to 52/h at 30 km/h with the AC off.

When the bus was moving and the windows were open, the ACH increased 21‐ to 59‐fold compared to when the windows were closed (Figure 3A–D). For example, when the bus was moving at 30 km/h, with mannequins, and the AC off, the rate increased from 2.5/h with all windows closed (Figure 3A) to 52/h with them fully open (Figure 3C), and when the bus was moving at 30 km/h, without mannequins, and the AC off, it increased from 2.5 /h with all windows closed (Figure 3B) to 72/h with them fully open (Figure 3D).

In Figure 3B (no mannequins, all windows closed), the ACH with the AC off was 7.1 /h in the stop & go test whereas it was 2.5/h when the bus was moving steadily at 30 km/h. In the same figure, the ACH with the AC on was 8.5/h in the stop & go test whereas it was 2.4/h when the bus was moving steadily at 30 km/h. In Figure 3D (no mannequins, windows fully open, the AC off), the ACH in the stop & go test (58/h) was not notably different from the ACH in the bus moving steadily at 30 km/h (72 /h).

When all windows were closed, the ACH did not differ between tests with the AC on or off. For example, when the bus was moving at 30 km/h, it was 2.5 or 2.3/h with mannequins, whether the AC was off or on (Figure 3A), and 2.5 or 2.4/h without mannequins, whether the AC was off or on (Figure 3B).

When all windows were closed, there were almost no differences in the ACH between tests with and without the mannequins. For example, at 30 km/h with the AC off, it was 2.5/h with mannequins and 2.5/h without mannequins, and at 30 km/h with the AC on, it was 2.3/h with mannequins and 2.4/h without mannequins (Figure 3A and B). However, when the bus was moving at 30 km/h with the windows fully open, the ACH was high (72/h) without mannequins and with the AC off, compared to 52/h with mannequins and the AC off (Figure 3C and D).

**FIGURE 4:**
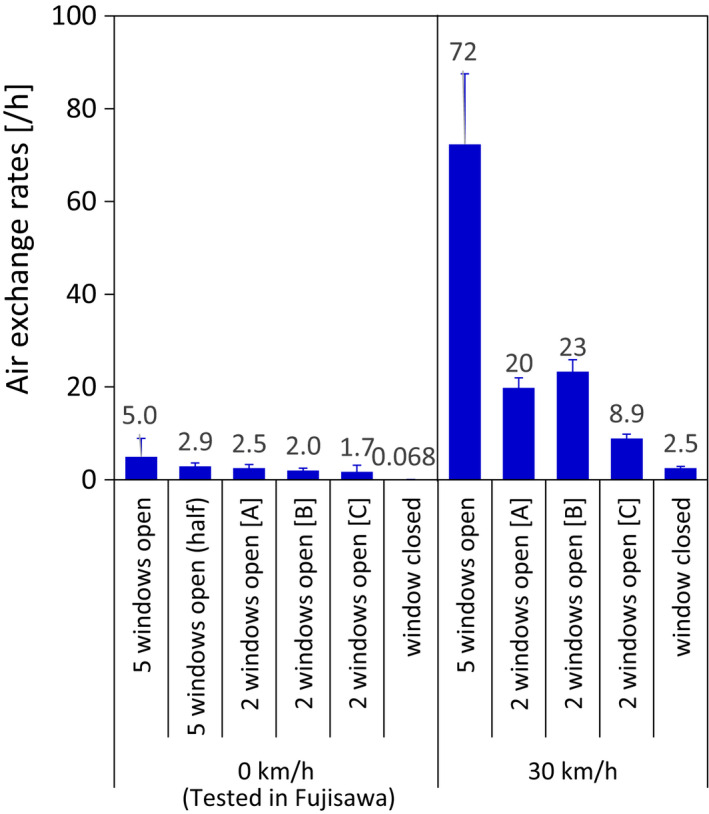
ACH in the bus without mannequins but with different numbers of open windows. The stationary tests were conducted in the car barn at the Fujisawa plant, and the moving tests were conducted outdoors at Tsukuba. (A) Right front and left rear windows open; (B) right front and right rear windows open; (C) right rear and left rear windows open

The ACH differed depending on the number of open windows and their locations (Figure 4). The ACH increased as the number of open windows increased: In the stationary bus, it increased from 0.068/h with the windows closed to 1.7–2.5/h (the range depending on the position of window‐opening) with two windows open and to 5.0/h with all five windows open. When the bus was moving at 30 km/h, the rate increased from 2.5/h with windows fully closed to 8.9–23/h (the range depending on the position of window‐opening) with two windows open, and to 74/h with five windows open. In the moving bus with two windows open, the ACH was higher when the two open windows were those on the right front and left rear (20/h; [A]) or the right front and right rear (23/h; [B]) than when they were those on the right rear and left rear (8.9/h; [C]).

**FIGURE 5:**
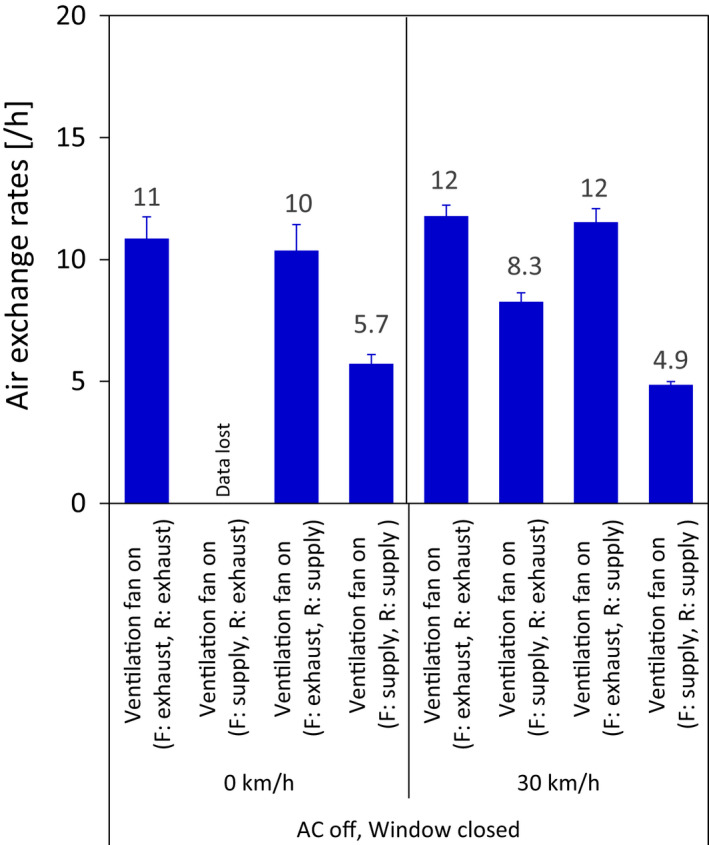
Effects of ventilation fan operation on the ACH in the bus without mannequins. F and R indicate the front and rear ventilation fan, respectively

Ventilation fan operation also affected the ACH when the windows were fully closed (Figure 5). That is, the ACH was 2.5/h at 30 km/h with the ventilation fan off (Figure 3A), while the ACH was 4.9–12/h (the range depending on the mode of the front and rear ventilation fan) at 30 km/h with the ventilation fan on (Figure 5). The ACH was higher when the front and rear fans were both set to exhaust mode (11/h at 0 km/h and 12/h at 30 km/h), as well as when the front and rear fans were set to exhaust and supply mode, respectively (10/h at 0 km/h and 12/h at 30 km/h), than when both the front and rear fans were set to supply mode (5.7/h at 0 km/h and 4.9/h at 30 km/h). On the contrary, when the bus was moving with the windows fully open, ventilation fan operation did not affect the ACH. That is, the ACHs were 36, 46, and 52/h at 10, 20, and 30 km/h, respectively, with the ventilation fans off (Figure 3C), while the ACHs were 20, 46, and 53/h at 10, 20, and 30 km/h, respectively, with the ventilation fans on [front set to supply and rear set to exhaust] (Figure S4).

### 3.2 Advection–diffusion of CO_2_ and particles

The CO_2_ concentration changes over 15 min in each seat are visualized in movies (Movies S1–S3). When the AC was off, CO_2_ emitted from the right rear seat spread forward via the center aisle, except when the rear ventilation fan was set to exhaust mode. When the rear ventilation fan was set to exhaust mode, it spread across to the opposite side of the bus and then forward. CO_2_ emitted from the front standing position spread toward the windows and then was reflected back and spread in the direction opposite to the emission direction. There was a noticeable tendency for the air to flow toward the front of the bus when the front fan was set to exhaust and the rear fan was set to supply, and for the air to flow toward the back of the bus when the front fan was set to supply and the rear fan was set to exhaust.

**FIGURE 6:**
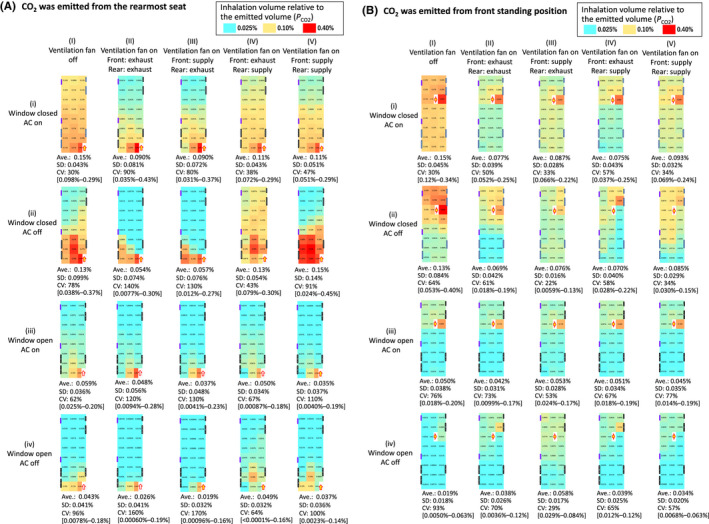
(A) Inhalation volume of CO_2_ relative to the volume emitted in the right rearmost part of the bus (*P*_CO2_), in stationary tests with mannequins. Average of surrounded data was used for two points whose data were lost. (B) Inhalation volume of CO_2_ relative to the volume emitted in the front center part (standing) of the bus (*P*_CO2_), in stationary tests with mannequins. Average of surrounded data was used for a point whose data were lost

In stationary tests with mannequins, CO_2_ concentration was higher near the source and lower away from the source under all conditions (Figure 6), but the CO_2_ concentration tended to be reduced overall when windows were open (rows i → iii, ii → iv) or the ventilation fans were on (columns I → II–IV). When CO_2_ was emitted at the rearmost seat with the windows closed (the upper two rows (i and ii) in Figure 6A), the CO_2_ concentration varied more by location with the AC off (coefficient of variation [CV] 43–140% (row ii)) than when the AC was on (CV 30–90% (row i)). When CO_2_ was emitted at the front standing position with the windows closed (Figure 6B), the variation of the CO_2_ concentration by location differed little between AC off (CV 22–64% (row ii)) and AC on (CV 30–57% (row i)), although concentrations in the seats in front of the emission source were higher when the AC was on (0.22–0.25% (row i and columns II–V)) than when the AC was off (0.13–0.22% (row ii and columns II–V)), unless the ventilation fan was off.

**FIGURE 7:**
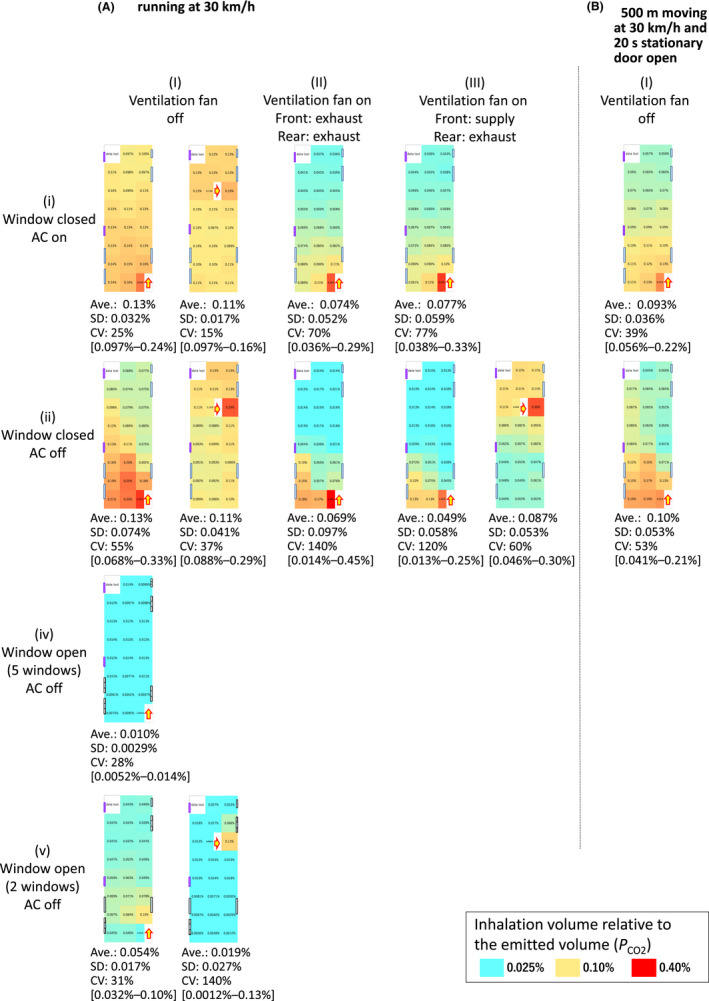
Inhalation volume of CO_2_ relative to the volume emitted at the positions indicated by arrows (*P*_CO2_) in moving tests with mannequins

In stationary tests with mannequins, the average CO_2_ concentration in the bus was reduced by 23–86% (average 55%) when the windows were open compared to when they were closed (Figure 6, rows i → iii and ii →iv). For example, when the ventilation fan was off and the AC was on, *P*_CO2_ decreased by 39% from an average of 0.15% when the window was open (row i column I) to an average of 0.059% when the window was closed (row iii column I). When the windows were closed, the in‐bus concentration did not differ between a moving (30 km/h, Figure 7A rows i, ii and columns I–III) and stationary bus (Figure 6A, B rows i, ii and columns I–III), but when the windows were open, the indoor concentration was reduced by 76% in the moving bus at 30 km/h with the AC off (0.010% of *P*_CO2_, Figure 7A row iv and column I) compared to that in the stationary bus with the AC off (0.043% of *P*_CO2_, Figure 7A row iv and column I). In addition, the CO_2_ concentration in the bus in the stop & go tests with the windows closed (0.093 and 0.10% of *P*_CO2_, Figure 7B rows i, ii and column I) was decreased by 26% and 27% compared to that in the moving bus at 30 km/h with the windows closed (0.13 and 0.13% of *P*_CO2_, Figure 7A rows i, ii and column I).

**FIGURE 8:**
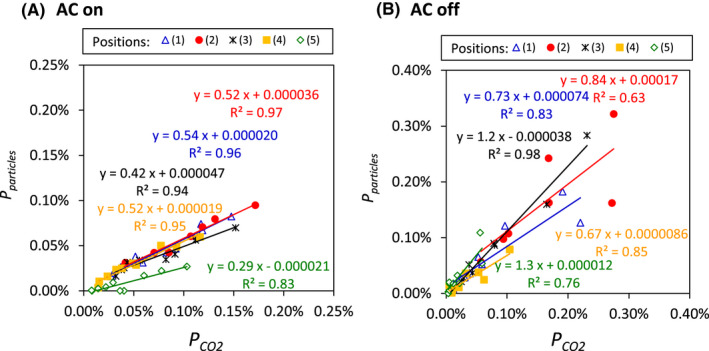
Relationships between *P*_CO2_ and *P*_particles_ in the bus at five measurement positions with the AC (A) on and (B) off

Concentration profiles for particles on positions and window open or closed were similar to those for CO_2_, that is, higher at points (1)–(3) than (4) and (5) and higher with the windows open than with the windows closed (Figure S5). However, the concentration trends for particles on AC operation was different from CO_2_, that is, lower with the AC on than with the AC off (Figure S5). With the AC off, *P*_CO2_ showed an approximately 1:1 correlation with *P*_particles_ (Figure 8B), whereas with the AC on (Figure 8A), the slope of the *P*_particle_–*P*_CO2_ relationship was approximately 0.5. With the AC on, the slope of *P*_particle_–*P*_CO2_ relationship was smallest at point (5) which is the only point farther from the source than the AC suction inlets (Figure 8A).

## 4 DISCUSSION

When the bus was moving continuously without stopping and opening the door with the windows closed and the ventilation fan not operating, ACH ranged from 0.78/h (10 km/h) to 2.5/h (30 km/h) (Figure 3A and B). However, when the bus was moving as it would simulate during normal route bus operations (stop & go tests: repeatedly moving at 30 km/h for 500 m, and then stopping and opening the door for 20 s), the ACH increased to 7.1/h (AC off) and to 8.5/h (AC on) (Figure 3B). By comparison, ACH in actual school buses under normal operating conditions (2.60–4.55/h)^23^ were less than the values obtained during the stop & go tests in this study, but more than those obtained when the bus was moving steadily. In the study by Rim et al,^23^ the door was open for a time period about 1/9 as long as the period that the bus was moving, whereas in this study the door was open for a period only about 1/3 of the period. This result implies that opening the door while the bus is stopped increases the ACH. However, when the bus was moving with the windows open or the ventilation fans on, the ACH was not increased by stopping and opening the door. These results suggest that when the windows are closed or the ventilation fan is off, the ACH can be increased by extending the time that the bus is stopped with the door open, but when the windows are open or the ventilation fan is on, stopping and opening the door has no effect.

The ACH increased as the speed of the bus increased, whether the windows were closed or open (Figure 3). The same trends have been observed in school buses^23^ and in subway trains.^26^ This result can be explained by the fact that, as the speed of the bus increases, the difference in air pressure between inside and outside the car increases, thereby promoting ventilation. In this study, we measured the ACH at a maximum speed of 30 km/h. Shinohara et al^26^ observed an ACH of 3.1/h in a closed train car moving at 53 km/h with a gap area/floor area ratio (C‐value; airtightness performance in accordance with Japanese Industrial Standards)^32^ of 5.4 cm^2^/m^2^, whereas the C‐value of the bus used in this study was 8.0 cm^2^/m^2^. Therefore, it can be assumed that the ACH would increase further as the bus speed increased above 30 km/h. However, further research is required on the relationships between C‐value and ACH and between ACH and speed.

The average vehicle speed of route buses varies greatly among urban, suburban, and rural areas, and between commuting hours and other times of the day. Therefore, not only the number of passengers and the distance between bus stops but also the average speed of the vehicle, which varies depending on the location and time of day, must be taken into consideration when implementing infection control countermeasures. In addition, because the ACH varies with vehicle speed, it is desirable to ensure a certain level of ACH regardless of speed. Therefore, buses should be equipped with a ventilation system with a capacity sufficient to achieve the desire ACH, and/or the AC system should be equipped with filters that can reduce the concentration of airborne droplet nuclei. Further research is needed to assess risk reduction by filters.

Ventilation fan operation (front, supply; rear, exhaust) had no effect when the windows were open and the bus was moving (compare Figure 3 (C) and Figure S4). This suggested that when the bus was moving with the windows open, the air exchange due to the windows being open greatly exceeded the ventilation fan capacity; as a result, the ventilation fan acted only as another aperture.

Each of the ventilation fans installed in this bus has a capacity of 530 m^3^/h; thus, the maximum ACH during fan operation for exhausts settings was 21/h. In fact, the ACH did not differ notably between stationary and moving (30 km/h) tests when the settings of the front and rear fans were the same: both set to exhaust, 11 and 12/h, respectively; front set to exhaust and rear set to supply, 10 and 12/h, respectively; and both set to supply, 5.7 and 4.9/h, respectively (Figure 5). The decrease in the airflow volume during actual operation compared to the ventilation fan capacity might be due to a decrease in the pressure difference between the inside and outside of the bus (i.e., resistance to air being pressed/sucked in via gaps in the exterior surface of the bus). In general, pressure on the roofs of high buildings is negative relative to the atmospheric pressure,^33^ and a simulation result has suggested that when a bus is moving, pressure on the roof of the bus will also be negative relative to the surrounding air pressure.^34^ Unless there is a strong wind, the wind speed above the roof of a bus should not be high unlike a high building, so pressure on the bus roof is not negative when the bus is stationary. However, in this study, the ACH did not differ between the stationary and moving tests with the ventilation fans on; this result suggests that the contribution to ventilation of negative pressure on the roof is small, even when the bus is moving, and the contribution of static pressure inside the bus is large. The ACH was greater when both front and rear fans were set on exhaust than when both fans were set on supply because of characteristic P‐Q curves (where P is static pressure and Q is the airflow rate) of the fans, which we obtained from the manufacturer (data not shown); namely, the airflow rate in the exhaust direction becomes greater than that in the supply direction for any static pressure value above zero. As a result, when both the front and rear fans were set on exhaust, the static pressure in the bus (calculated to be 28 Pa) was higher than that when both front and rear fans were set on supply (calculated to be 14–15 Pa). This suggests that the difference in the ACH between air supply and exhaust was due to the difference in the static pressure‐air volume characteristics of the fans installed in this bus between exhaust and supply due to the fan's design.

The more windows were open in the bus, the greater ACH was observed. The same trend was observed on subway trains in the present study.^26^ While the bus was moving with two windows open, the ACH was greater when one front and one rear window was open than when both rear windows (on opposite sides of the bus) were open. We attribute this result to the fact that, when the bus is moving, air entering through a front window can escape via a rear window, but when the open windows are opposite each other, it is harder for air entering via one window to escape via the other.

The ACH values obtained in this study were 20–72/h when the bus was continuously moving with the windows open and 7.1–8.5/h when the bus was moving and repeatedly stopping and opening doors with the windows closed as during normal route bus operation. Dai and Zhao,^22^ who applied the Wells–Riley equation to estimate the risk of airborne transmission of COVID‐19 in confined spaces, showed that to keep the risk of infection in a bus with one infected person to less than 0.1% and 0.5%, an ACH of 7.0–24/h and 1.4–4.8/h, respectively, was sufficient, provided that the passengers wear masks. Therefore, the infection risk under the conditions in our study would sometimes be above 0.1% but would always be lower than 0.5% if the passengers wore masks.

When the windows were closed and the ventilation fans were off and CO_2_ was emitted from a single location and spread in the bus by advection and diffusion, the maximum *P*_CO2_ near the source was 0.29–0.40% if the bus was stationary and 0.16–0.33% if it was moving, whereas the average *P*_CO2_ in the entire vehicle was 0.13–0.15% if the bus was stationary and 0.11–0.13% if it was moving. *P*_CO2_ inside the bus calculated during the first 15 min of CO_2_ emission by using ACH with the windows closed and ventilation fans off (0.34–0.87/h stationary and 2.3–2.5 /h moving at 30 km/h) under the assumption of complete mixing was 0.14–0.15% in the stationary bus and 0.12% in the moving bus. Thus, exposure near the infected person was up to three times higher than after complete mixing, and the average exposure inside the bus was comparable to the results calculated by using ACH and assuming complete mixing.

**FIGURE 9:**
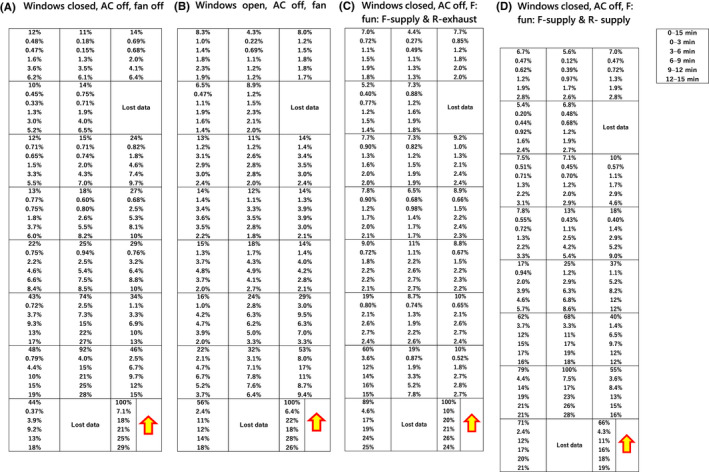
Normalized exposure data in the stationary city bus with (A) windows closed, AC off, fan off, (B) windows open, AC off, fan off, (C) windows closed, AC off, fan supply (front) & exhaust (rear), and (D) windows closed, AC off, fan supply (front) & supply (rear)

The CO_2_ emitted from the source spreads along the emitted stream forming a plume; then, beyond a certain distance, the CO_2_ spread to the surrounding area via other airflows and advection–diffusion, which obscure the plume and increase the overall concentration in the surrounding area. The exposure to CO_2_ in the plume can be managed by distancing and controlling airflow, and the CO_2_ that has spread to the surrounding area can be managed by filtration and ventilation. Following Drivas et al (1996), we calculated normalized exposure for each seat at 3‐min intervals over 15‐min period (0–3, 3–6, 6–9, 9–12, and 12–15 min) and for the total 15‐min period (Figure 9). The CO_2_ exposure at seat where CO_2_ exposure was maximum in the bus during the 15 min was fixed to 100%, and then, the CO_2_ exposure at other seats and for other time durations was calculated relative to that maximum. The results showed that even when the AC was turned off, a plume could be detected only within about three seats from the source (Figure S6).

The correlation between *P*_particles_ and *P*_CO2_ when CO_2_ and particles were emitted at a single location was about 1:1 when the AC was off (Figure 8B), but when the AC was on, *P*_particles_ was lower than *P*_CO2_ although they were correlated (Figure 8A). In particular, when the AC was on, the ratio of *P*_particles_ to *P*_CO2_ at point (5) (see Figure 1C), the farthest point from the particle source, was lower than the ratio at points (1)–(4). This result implies that when the AC is on, some particles are removed from the air by being deposited on the wall because of the wind from AC or trapped in the AC dust filter. Further investigation of particle deposition on walls and collection on filters is required.

We used the following modified version of the Wells–Riley equation to calculate the risk of COVID‐19 infection, *R* [%], when there was one infected person in the bus.

$$R=\left(\text{1 ‐ exp}\left(‐qP_{\text{CO2}}t\right)\right)\times 100$$

Here, *R* [%] represents the probability that a passenger will be infected when one infected person is on the bus, *q* [/h] is the quanta generation rate, and *t* [h] is the ride time. We set *q* to 20 [/h], based on a previous report,^35^ for *P*_CO2_ we used the value calculated when the AC was off, when a 1:1 correlation with *P*_particles_ was observed; and for *t* we used 0.25 h. Since *R* can be approximated by *q*×*P*_CO2_ when *P*_CO2_≪1, the reduction rate of *R* is almost the same as the reduction rate of *P*_CO2_. The results showed that both the infection risk and *P*_CO2_ in the stationary bus were reduced by 23–86% (average, 55%) when the windows were open (*R*: 0.095–0.29%; *P*_CO2_: 0.019–0.059%) compared to the values with the windows closed (*R*: 0.27–0.76%; *P*_CO2_: 0.054–0.15%). When the bus was moving with the ventilation fans off, both the infection risk and *P*_CO2_ were reduced by 92% if the windows were open (*R*: 0.052%; *P*_CO2_: 0.010%) compared to the values with the windows closed (*R*: 0.67%; *P*_CO2_: 0.13%). A sensitivity analysis performed using previously reported quanta generation rates (14–48/h^22^; 0.37/h (resting, oral breathing) to 32/h (light activity, singing or speaking loudly), 50th percentile values^36^) showed almost the same risk reduction values: 23–86% (average, 55%) for a quanta generation rate of 0.37/h versus 23–86% (average, 55%) for a quanta generation rate of 48/h. This result confirmed that the risk was greatly reduced when the windows were open. When the ventilation fans were running and both the front and rear fans were set on exhaust, the risk was reduced by 38–57% (average, 48%) and 18–40% (average, 29%) with windows open and closed, respectively, when the bus was stationary, and it was reduced by 20–49% (average, 35%) when the bus was moving with windows closed, regardless of the location of the source. However, the risk can be increased in some cases when the front fan was set to exhaust and the rear fan was set to supply; thus, when ventilation fans are used to reduce the risk of infection in buses, both front and rear fans should be set to exhaust.

For comparison, we also calculated the infection risk of measles, which is much more contagious than COVID‐19. Stephens reported the quanta generation rates of measles were between ~570 and ~5600/h.^37^ The infection risk of measles in the stationary bus used in the present study was estimated to be reduced between ~22% and ~84% (average, 54%) for ~570 of quanta generation rate and between ~14% and ~72% (average, ~40%) for ~5600/h of quanta generation rate, when the windows were open (*R*: between ~2.7% and ~8.0% for ~570/h and between ~23% and ~56% for ~5600/h) compared to the risk with the windows closed (*R*: between ~7.5% and ~19% for ~570 /h and between ~53% and ~88% for ~5600/h). Although the reduction in risk of infection achieved by opening the windows is not much different from that for COVID‐19, additional countermeasures are required for vehicles where measles infection is a concern because of its high infection risk.

It has been suggested that a SARS‐CoV‐2 transmission cluster in a tourist bus in China may have been caused by airborne transmission to seats distant from the infected person via AC air circulation.^18^ Because the bus used in the present study had many AC outlets (air supply vents) above the passengers, it would be possible for the virus to be spread from the infected person to other areas of the bus by the AC air circulation. However, we found that when the AC was on, the average CO_2_ exposure concentration sometimes increased and sometimes decreased, depending on other conditions, but the average particle exposure concentration was decreased to approximately less than half; this result suggests that filtration through even a coarse dust filter could reduce the infection risk when the AC is on. By improving the performance of the AC filters, it might be possible to further reduce the risk. Furthermore, the present study results suggest that the risk was higher at locations between the AC inlet and the infected person (points (1)–(4) in Figure 1) than at locations beyond the AC inlet from the infected person (point (5)). This result indicates that the risk could be further reduced by having the AC inlets at multiple locations in the bus rather than at a single location.

However, when CO_2_ and particles were emitted at the level of a standing passenger, the exposure concentration in the seat in front of the emission location tended to be higher when the AC was on, possibly owing to the effect of downward airflow from the AC. Thus, the degree of risk may depend on how droplet nuclei and microdroplet are spread in the bus by the AC airflow, regardless of which seat the infected person sits in. Therefore, further study of how AC circulation and particle removal by filters affect exposure is necessary.

The route bus used in this study was the standard type produced by the manufacturer (Isuzu Motors, Ltd.) with a 70% share of the market for route buses in Japan in 2019, including production by the original equipment manufacturer (Hino Motors, Ltd.),^38^ although the position and number of openable windows vary from bus to bus. Those route buses with a greater number of openable windows may have larger ACHs than the bus used in this study. All of the buses built by this bus manufacturer are equipped with ventilation fans and AC system produced by a single manufacturer (Denso Co.). Thus, the fans and AC system of all buses from this bus manufacturer are basically similar in structure and performance. In addition, more than half of the buses built by the manufacturers (Mitsubishi Fuso Truck and Bus Co.) with the remaining 28% share of the Japanese market also use Denso Co. ventilation equipment. Therefore, not only the qualitative results (the relationship between the number/position of window openings and ACH, and the overall homogenization of air by AC operation) but also the quantitative results (the determined ACHs) obtained in this study may be similar to those for other route buses used in Japan. In route buses used in other countries, however, ACHs might vary more, depending on the size/position/number of openable windows, body structure, and the ventilation fan and AC capacities of buses used.

There is both temporal and spatial uncertainty in the results of the measurement of CO_2_/particle spread in the bus. They are caused by variance/error in CO_2_/particle emission/measurement and variance of airflow turbulence. Although it is possible to reduce the uncertainty by the repeated measurements, the test was conducted in a single session for each condition in the present research since it was hard to conduct multiple tests in this study according to the restrictions of bus usage and location. In addition, although the air exchange rates can vary depending on the outdoor air conditions (indoor–outdoor temperature difference, outdoor wind speed, etc.), these effects were also not evaluated in this study. The coefficient of variation (CV) of CO_2_ and particle emission during the advection–diffusion in test in this study was 0.85% ± 0.51% and 8.4% ± 3.7% (mean ± SD), respectively. The CV of the instrumental error was 0.37% (7000 ppm) to 6.8% (450 ppm) and 2.3% ± 2.2% for CO_2_ and particle measurement, respectively. Although the eddy generated by the airflow from the emission source might be randomly generated, the uncertainty cannot be evaluated in the present measurement. It is necessary to evaluate the uncertainty of the results in the present research by simulating the behavior of CO_2_/particles with CFD simulations in the future.

## 5 CONCLUSION

The ACH increased when the number of open windows increased and as vehicle speed increased. When the windows were open and the bus was moving, operation of the ventilation fans made no difference. Therefore, it is not necessary to operate ventilation fans while a bus is moving with windows open. While the bus was moving with the windows closed, it was effective to operate the ventilation fans by setting both the front and rear fans to exhaust or by setting the front fan to exhaust and the rear fan to supply. We confirmed that opening windows greatly reduces the risk of infection (92% in a moving bus), and that when the windows are closed, the risk of infection can be reduced by setting both front and rear ventilation fans to exhaust (35% in a moving bus). Although the risk is possible to be reduced by using even a coarse dust filter, a better‐performing filter can be effective in reducing risk, especially when the AC is to be operated.

## CONFLICT OF INTEREST

The authors have no conflict of interest to declare.

## AUTHOR CONTRIBUTIONS

N.S.: Conceptualization, Methodology, Investigation, Data analysis, Writing‐original draft, Writing‐review & editing; K.T.: Conceptualization, Investigation, Writing‐review & editing; K.N. Methodology, Investigation, Writing‐review & editing; H.K.: Methodology, Investigation, Writing‐review & editing; J.S.: Investigation, Writing‐review & editing; I.O.: Investigation, Writing‐review & editing; Y.M.: Quality assurance, Writing‐review & editing; H.S.: Quality assurance, Writing‐review & editing; W.N. Investigation, Writing‐review & editing.

## Supporting information

Supplementary Material
