# Supplementary material for: Air exchange rates and advection–diffusion of CO2 and aerosols in a route bus for evaluation of infection risk
Source: Indoor Air. 2022 Mar 20;32(3):e13019. doi: 10.1111/ina.13019 (PMC9111735; doi:10.1111/ina.13019)
Supplement: Supplementary file 1 — Supplementary Material [file INA-32-0-s001.docx]

**DOI: 10.1111/ina.13019**

**SUPPLEMENTARY MATERIALS**

**FOR**

**Air Exchange Rates and Advection-Diffusion of CO_2_ and Aerosols in a Route Bus for Evaluation of Infection risk**

Naohide Shinohara^*,1^, Koichi Tatsu^2^, Naoki Kagi^4^, Hoon Kim^3^, Jun Sakaguchi^2^,

Isamu Ogura^1^, Yoshiko Murashima^1^, Hiromu Sakurai^1^, Wataru Naito^1^

^1^ National Institute of Advanced Industrial Science and Technology (AIST), 16-1 Onogawa, Tsukuba, Ibaraki 305-8569, Japan

^2^ Isuzu Motors Ltd., 8 Tsuchidana, Fujisawa, Kanagawa 252-8501, Japan

^3^ National Institute of Public Health, 2-3-6 Minami, Wako, Saitama 351-0197, Japan

^4^ Tokyo Institute of Technology, 2-12-1 Ookayama, Meguro-ku, Tokyo 152-8552, Japan

^5^ University of Niigata Prefecture, 471 Ebigase, Higashi-ku, Niigata- City, Niigata 950-8680, Japan

 * Correspondence to: Naohide Shinohara; e-mail: [n-shinohara@aist.go.jp](mailto:n-shinohara@aist.go.jp);

TEL: +81-29-861-8030, FAX: +81-29-861-8411

**↑Double click please↑**

**↑Double click please↑**

**↑Double click please↑**

**Figure S1 The commuter bus used in this study**

**Figure S2 Photographs of a bus window, a ventilation fan, and AC inlet and outlet vents**

**Figure S3 Schematic diagram of a sliding bus window with two panes.**

**Figure S4 ACH when the bus was moving with mannequins, the windows fully open, the AC off, and the ventilation fans on (front, supply; rear, exhaust).**

**Figure S5 Number of inhaled particles relative to the number emitted in the right rearmost part of the bus (Pparticles). F, front; R, rear.**

**Figure S6-1. Normalized exposure data emitted from rearmost right seat in the stationary city bus with**

**(a) windows closed, AC off, fan off, (b) windows open, AC off, fan off, (c) windows closed, AC off, fan supply (front) & exhaust (rear), and (d) windows closed, AC off, fan supply (front) & supply (rear).**

**Figure S6-2. Normalized exposure data emitted from front standing point in the stationary city bus with (a) windows closed, AC off, fan off, (b) windows open, AC off, fan off, (c) windows closed, AC off, fan supply (front) & exhaust (rear), and (d) windows closed, AC off, fan supply (front) & supply (rear).**

**Table S1. List of the experiment of air exchange rates**

**Table S2. List of the experiment of advective-diffusion of artificial nuclei**

**Table S3. Data on air exchange rates in 24 seats in every test**
